# Anaemia in Pregnancy Is Associated with Advanced HIV Disease

**DOI:** 10.1371/journal.pone.0106103

**Published:** 2014-09-15

**Authors:** Vikesh Nandlal, Dhayendre Moodley, Anneke Grobler, Jayanthilall Bagratee, Niren R. Maharaj, Paul Richardson

**Affiliations:** 1 Department of Obstetrics and Gynaecology, Nelson R Mandela School of Medicine, University of KwaZulu Natal, Durban, South Africa; 2 Department of Pathology, Johns Hopkins University School of Medicine, Baltimore, Maryland, United States of America; 3 Center for the AIDS Programme of Research in South Africa-CAPRISA, Durban, South Africa; UCL Institute of Child Health, University College London, United Kingdom

## Abstract

**Background:**

Anaemia is a common clinical finding in HIV infected women and has been associated with advanced disease. The use of antiretroviral drugs such as Zidovudine (ZDV) either for prevention of mother to child transmission (MTCT) of HIV or used in combination with other antiretrovirals have been implicated in the development or increased severity of anaemia. We report the prevalence, type, severity and incidence of anaemia in a cohort of HIV infected women who initiated antiretroviral prophylaxis or treatment during pregnancy.

**Methods and Materials:**

This is a retrospective cohort data analysis of 408 HIV infected pregnant women who participated in a breastfeeding intervention study (HPTN 046 Study, ClinicalTrials.gov NCT 00074412) in South Africa. Women initiated zidovudine prophylaxis for PMTCT or triple antiretroviral treatment in pregnancy according to the standard of care. Laboratory and clinical data in pregnancy, <72 hours and 2 weeks postdelivery were extracted from the main database and analysed.

**Results:**

The mean Hb concentration was 10.6 g/dL at baseline and 262/408 (64.2%) women were diagnosed with anaemia (Hb<11 g/dL) in pregnancy, 48/146 (32.9%) subsequently developed anaemia intrapartum or postpartum and 89/310 (28.7%) of all cases of anaemia remained unresolved by 2 weeks postdelivery. In a univariate analysis, CD4 count and gravidity were significant risk factors for anaemia in pregnancy, RR 1.41; 1.23–1.61 (p<0.001) and 1.10; 1.01–1.18 (p = 0.02) respectively. After adjusting for antiretroviral regimen, age and gravidity in a multivariable analysis, only the CD4 count remains a significant risk factor for anaemia in pregnancy and postdelivery.

**Conclusion:**

In conclusion, anaemia was most common among women in the advanced stage of HIV infection (CD4<200 cells/mm^3^). There was no evidence of an association between ZDV or triple ARVs and anaemia.

## Introduction

The World Health Organization estimates that nearly half of all women in low resource countries have anaemia (52%) [1]. Women of reproductive age (15–49 years) are most commonly affected and likely due to excessive blood loss during menstruation, insufficient dietary intake, parasitic infections and increased physiological demand during pregnancy [2]. In pregnancy, iron deficiency anaemia is associated with poor birth outcomes such as increased risk of still births, low birth weight infants, intrauterine growth restriction and neonatal sepsis [3], [4].

More recently, anaemia was also a common clinical finding in HIV infected women and has been associated with advanced disease. In Sub-Saharan Africa, a region most affected by the HIV pandemic, anaemia in HIV infected women has been independently associated with adverse maternal and fetal outcomes in pregnancy [5]–[7]. In 2012, the Saving Mothers Report in South Africa concluded that HIV infection (70%) and anaemia (30%) were the commonest conditions among women who died during pregnancy or in the puerperium [8]. Although not explored in the Saving Mothers Report, through other studies the use of antiretroviral drugs such as Zidovudine (ZDV) either for prevention of mother to child transmission (MTCT) of HIV or used in combination with other antiretrovirals have been implicated in the development or increased severity of anaemia [9]. A recent clinical trial across three Sub-Saharan African countries demonstrated that women randomized to short or longer ZDV containing regimens were at similar risk of severe anaemia and the longer the duration of a triple antiretroviral (ARV) regimen the greater the reduction in the incidence of anaemia [10]. These findings are inconsistent with studies conducted in Malawi and Mozambique that implicated ZDV containing regimens as risk for anaemia [11].

Zidovudine containing regimens as prophylaxis for PMTCT remains the most affordable and cost-effective option in most Sub-Saharan African countries with a high HIV disease burden. Yet, not many studies have sought to confirm if anaemia, a potential side effect of Zidovudine use, could be attributed to the HIV disease stage itself and could be avoided if pregnant women in the advanced stage of disease are treated with a triple antiretroviral regimen.

We report the prevalence, type, severity and subsequent incidence of anaemia in a cohort of HIV infected women during pregnancy, within 72 hours post-delivery and 2 weeks post-delivery in association with HIV disease stage (CD4 count) alone or in relation to ARV prophylaxis or treatment. HIV positive pregnant women in South Africa during the course of the HPTN046 study were eligible for triple ARV (D4T/3TC/NVP) if they were in the advanced stage of the disease (CD4<200) and ZDV used as prophylaxis to prevent MTCT in women not meeting the treatment criteria. We also describe the potential impact of anaemia on maternal and infant birth outcomes.

## Methods

### Ethics Statement

This secondary data analysis study was reviewed and approved by the Institutional Review Board of University of KwaZulu Natal. Participant informed consent specific to this sub study was not obtained; hence participant records were anonymized and de-identified prior to analysis.

This study is a retrospective cohort data analysis of mother-infant pairs who participated in the HPTN 046 Study (Clinical Trials.gov. Identifier: NCT 00074412) conducted in Umlazi, South Africa [12]. The HPTN046 Study was a Phase III randomized controlled trial conducted in South Africa, Uganda, Zimbabwe and Tanzania to determine the efficacy and safety of an extended regimen of nevirapine in infants born to HIV-infected women to prevent transmission of HIV through breastfeeding. Further details of the study conduct and outcome measures have previously been published and can also be accessed at www.HPTN.org. Data for the South African cohort prior to randomisation in the clinical trial were captured on site and therefore readily available for this sub study analysis. For this reason, other sites were not included.

The study was conducted at a clinical trial research facility based at a tertiary hospital in Umlazi, South Africa. Umlazi is an urban township with an antenatal HIV prevalence of 40%. HIV positive pregnant women, 18 years or older, intending to breastfeed, free from any serious medical complication and residing in Umlazi were referred from the primary health care clinics in Umlazi to the research facility.

The women initiated either ZDV prophylaxis or triple ARV (D4T/3TC/NVP) at the primary health care clinic prior to being referred for study participation at the clinical research site [13]. Women were enrolled at an average gestational age of 28 weeks between February, 2007 and March, 2010. After enrollment, study visits occurred within 72 hours post-delivery and at two, five and six weeks, and at three, six, and 12 months postpartum. At each visit a medical history and physical examination were completed. HIV disease staging was completed using the WHO staging system. A complete blood count (CBC) and CD4+ lymphocyte count was obtained in a local laboratory (BARC, Johannesburg) certified by the NIAID Division of AIDS Quality Assurance Program. Haematological results were reported with age-related reference intervals for the South African population. Evaluations at delivery included birth outcomes, mode of delivery and obstetrical complications. Clinical evaluations of the infant included a physical examination and a CBC within 72 hours of birth, two and five weeks of age and at subsequent follow-up visits up to 18 months of age. This sub study utilised data obtained at enrolment in pregnancy, within 72 hours of delivery and two weeks post-delivery. Women also received the standard combination of micronutrient supplementation (iron, folate and multivitamins) during pregnancy [14].

#### Definitions

Anaemia in women was characterised hemoglobin (Hb) levels below 11 g/dL and severity of Anaemia was graded as follows: grade 1 or mild (9.5–10.5 g/dL), grade 2 or moderate (8–9.4 g/dL), grade 3 or severe (6.5–7.9 g/dL) and grade 4 or life threatening (<6.5 g/dL). Anaemia in the newborns was defined as hemoglobin (Hb) levels below 13 g/dL. Severity of anaemia in the newborns was graded as follows: grade 1 or mild (12–13 g/dL), grade 2 or moderate (10–11.9 g/dL), grade 3 or severe (9–9.9 g/dL) and grade 4 or life threatening (<9 g/dL).

Pre-existing cases of anaemia at baseline in pregnancy were included in determining the prevalence of anaemia in pregnancy. New cases of anaemia were labelled as incident anaemia in women who first developed anaemia (Hb<11.0 g/dL) at delivery or 2 weeks post-delivery. Resolution of anaemia was defined as recovery in Hb level to ≥11.0 g/dL at delivery or 2 weeks post-delivery.

### Post-hoc Power Estimation

If the proportions of pregnant women with a higher CD4 count (>200 cells/mm^3^) receiving ZDV or D4T/3TC/NVP and who have anaemia were 0.6 and 0.42 respectively then one would have 80% power to detect a difference between the two groups if there were 200 women in the ZDV group and 100 women in the D4T/3TC/NVP group. In the lower CD4 count group (<200 cells/mm^3^), having 30 women per group would have 80% power to detect a difference between the two groups if the proportions of women with anaemia were 0.55 and 0.9, respectively.

Data were analysed using EpiInfo version 6.0 and SAS version 9.3. Maternal and infant characteristics were summarized using means, proportions, and 95% confidence intervals where applicable.

Relative risks and 95% confidence intervals were calculated to determine risk factors for anaemia. P-values were calculated using Fisher's exact test or chi-square tests. Multivariable models were fitted to determine adjusted relative risks using a generalised linear approach with a log-binomial model. All variables were included in the multivariable analysis.

## Results

Four hundred and eight HIV positive pregnant women ranging between 18 and 42 years were included in this analysis. The majority (90.2%) was single mothers, 21.6% were primigravidae and women were screened for anaemia at a median gestational age of 32 weeks (IQR 30–35 weeks).

**Figure 1:**
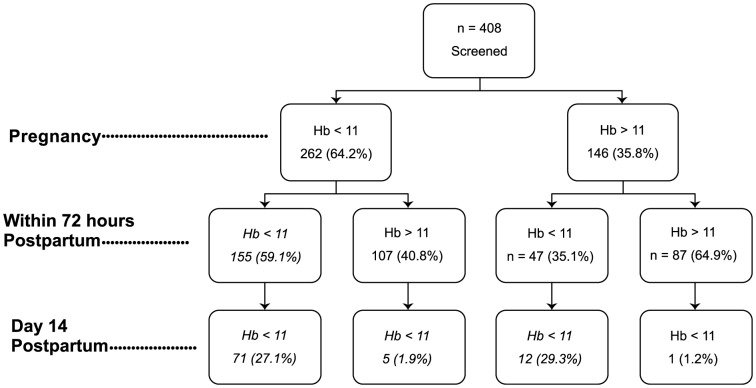
Prevalence, incidence and recovery of anaemia in pregnancy, within 72 hours postpartum and day 14 postdelivery.

The mean Hb concentration was 10.6 g/dL at baseline and 262/408 (64.2%) women were diagnosed with anaemia (Hb<11 g/dL) in pregnancy (prevalence), 48/146 (32.9%) subsequently developed anaemia intrapartum or postpartum and 89/310 (28.7%) of all cases of anaemia remained unresolved by 2 weeks post-delivery (Figure 1). A total number of 57 of the 408 (13.9%) women enrolled in pregnancy were missed at delivery (n = 30) or 2 weeks post-delivery (n = 27).

The median CD4 count among women with anaemia and without anaemia was 320 (IQR 217–463) and 392 (IQR 285–546) respectively. A significantly larger proportion of the 262 pregnant women with anaemia (56/262) were in an advanced stage of HIV disease (CD4<200) when compared to women without anaemia (10/146) (21.4% vs 6.9%; p<0.001).

Anaemia was predominantly mild during pregnancy (80.9%) and post-delivery (>75%), with the more severe cases of anaemia (Grade 3 and 4) presenting in 3.5% and 3.4% of the women immediately post-delivery and 2 weeks post-delivery respectively (Table 1). Normocytic normochromic anaemia was most commonly diagnosed in pregnancy (82.8%), within 72 hours of delivery (68.3%) and at 2 weeks postdelivery (62.9%). Furthermore, macrocytic anaemia also occurred in a third (30.4%) of the women who presented with persistent anaemia at 2 weeks post-delivery. Microcytic hypochromic anaemia remained least common in pregnancy (9.5%) and post-delivery (4.0–6.7%).

**Table 1:** Anaemia in pregnancy, within 72 hours of delivery and 2 weeks post delivery.

	Pregnancy N = 408	0–72 Hours Delivery N = 378	2 weeks Post Delivery N = 351
**Hb (g/dL)**			
Mean (Range)	10.6 (7.4–13.9)	10.4 (5.8–14.7)	11.8 (6.9–15.9)
Median (IQR)	10.6 (9.9–11.3)	10.9 (10.0–11.6)	11.9 (10.9–12.8)
**Prevalence of Anaemia** (Hb<11 g/dL) n(%)	262 (64.2)	202 (53.4)	89 (25.4)
**Severity of Anaemia n(%)**			
Grade 1 mild (9.5–10.5 g/dL)	212 (80.9)	156 (77.2)	71 (79.8)
Grade 2 moderate (8.0–9.4 g/dL)	47 (17.9)	39 (19.3)	15 (16.8)
Grade 3 severe (6.5–7.9 g/dL)	3 (1.2)	6 (3.0)	3 (3.4)
Grade 4 life threatening (<6.5 g/dL)	0	1 (0.5)	0
* **Types of Anaemia n(%)**			
Microcytic	25 (9.5)	8 (4.0)	6 (6.7)
Macrocytic	20 (7.7)	56 (27.7)	27 (30.4)
Normocytic	217 (82.8)	138 (68.3)	56 (62.9)

*Microcytic Anaemia: Mean Corpuscular Volume (MCV) <83fL.

Normocytic Anaemia: Mean Corpuscular Volume (MCV) 83–101 fL.

Macrocytic Anaemia: Mean Corpuscular Volume (MCV) >101 fL.

Approximately two thirds (268/408; 65.9%) of the study population experienced some form of morbidity during pregnancy and post-delivery, with urinary tract infections (94/407; 23.1%) and abnormal vaginal discharge characteristic of vaginal infections (80/407; 19.7%) being most commonly reported (Table 2). Complications in pregnancy were reported in 177/261 (67.8%) and 91/146 (62.3%) of women with and without anaemia respectively (p = 0.28). Urinary tract infections were highly common in women with anaemia, but not significantly higher when compared to women without anaemia (RR 1.46 p = 0.07).

**Table 2:** Obstetric and Infant Outcomes in Association with Anaemia in Pregnancy.

	Total	No Anaemia (Hb≥11 g/dL)	Anaemia (Hb<11 g/dL)	RR (95%CI) p Value
	n = 408	n = 146	n = 262	
**Complications in Pregnancy n (%)**	268/407 (65.9)	91/146 (62.3)	177/261 (67.8)	1.09 (0.94–1.27) p = 0.28
Antepartum Haemorrhage	6/407 (1.5)	2/146 (1.4)	4/261 (1.5)	1.12 (0.21–6.03) p = 1.00
PreEclampsia	22/407 (5.4)	8/146 (5.5)	14/261 (5.4)	0.98 (0.42–2.28) p = 1.00
Abnormal Vaginal Discharge/Infection	80/407 (19.7)	39/146 (26.7)	41/261 (15.7)	0.59 (0.40–0.87) p = 0.01
Urinary Tract Infections	94/407 (23.1)	26/146 (17.8)	68/261 (26.1)	1.46 (0.98–2.19) p = 0.07
Genital Warts	20/407 (4.9)	6/146 (4.1)	14/261 (5.4)	1.31 (0.51–3.32) p = 0.64
Preterm Delivery	53/405 (13.1)	21/146 (14.4)	32/259 (12.4)	0.86 (0.51–1.43) p = 0.65
**Infant Outcomes**				
Live birth n(%) Median birth weight (kg) (IQR)	402/408 (98.5) 3.1 (2.9–3.4)	144/146 (98.6) 3.2 (2.8–3.5)	258/262 (98.5) 3.1 (2.9–3.4)	0.99 (0.97–1.02) p = 1.00 p = 0.72
Birth weight <2.5 kg	25/376 (6.7)	8/132 (6.1)	17/244 (6.9)	1.15 (0.51–2.59) p = 0.83
Infant Anaemia <13 g/dl n (%)	26/375 (6.9)	5/131 (3.8)	21/244 (8.6)	2.25 (0.87–5.84) p = 0.09
Infant HIV Status 5 weeks n (%) DNA Positive DNA Negative Missing Data	17 (4.2) 360 (88.2) 31 (7.6)	6 (4.1) 129 (88.4) 11 (7.5)	11 (4.2) 231 (88.2) 20 (7.6)	0.98 (0.36–2.59) p = 1.00

The median birth weight of infants born to the HIV positive study population was 3.1 kg ranging from 1.2 to 4.5 kg. Twenty five newborns (6.7%) were of low birth weight (<2.5 kg); 8/132 (6.1%) born to women without anaemia and 17/244 (7.0%) women with anaemia (Table 2). Anaemia (Hb<13 g/dl) was more than twice as common in infants born to women with anaemia when compared to infants born to women without anaemia (RR2.25; p = 0.09).

Among the 408 pregnant women screened for anaemia, 259 (63.5%) were receiving the ZDV regimen and 122 (29.9%) were receiving the triple ARV regimen (D4T/3TC/NVP). Antiretroviral data was unavailable for 27 women, who were therefore excluded from this particular analysis. Anaemia was more common among women with a CD4<200 receiving ZDV (75.9%) or triple ARV (91.2%) as compared to women with a CD4>200 receiving ZDV (61.3%) or triple ARV (60.2%). However, in a stratified analysis of women in the two categorical HIV disease stage groups (CD4<200 and CD4>200) receiving either ZDV or triple ARV regimen, the risk for anaemia was not statistically significant for any particular group (Table 3).

**Table 3:** Anaemia in Association with Antiretroviral Use and HIV Disease Stage (CD4 Count <200 cells/mm^3^).

	CD4 ≤200 cells/mm^3^			CD4 >200 cells/mm^3^		
	ZDV	D4T/3TC/NVP		ZDV	D4T/3TC/NVP	
	n/N (%)	n/N (%)	RR (95% CI) p-value	n/N (%)	n/N (%)	RR (95% CI) p-value
Anaemia in pregnancy	22/29 (75.9)	31/34 (91.2)	0.83 (0.66; 1.05) 0.17	141/230 (61.3)	53/88 (60.2)	1.02 (0.83; 1.24) 0.90
Anaemia <72 hours after delivery	22/28 (78.6)	21/34 (61.8)	1.27 (0.92; 1.77) 0.18	116/227 (51.1)	41/86 (47.7)	1.07 (0.83; 1.38) 0.61
Anaemia 2 weeks post delivery	12/24 (50.0)	12/34 (35.3)	1.42 (0.77; 2.60) 0.29	45/211 (21.3)	20/82 (24.4)	0.87 (0.55; 1.39) 0.64
Infant Anaemia At Birth	7/28 (25.0)	1/34 (2.9)	8.5 (1.11; 65.0)	15/227 (6.6)	3/86 (3.5)	1.89 (0.56; 6.38)

In a univariate analysis, CD4 count and gravidity were significant risk factors for anaemia in pregnancy, RR 1.41; 1.23–1.61 (p<0.001) and 1.10; 1.01–1.18 (p = 0.02) respectively. After adjusting for antiretroviral regimen, age and gravidity in a multivariable analysis, only CD4 count remains a significant risk factor for anaemia in pregnancy and post-delivery (Table 4).

**Table 4:** Association between HIV Disease Stage and Anaemia in Pregnancy and Postdelivery.

	Univariate analysis		Multivariable analysis	
Anaemia in pregnancy	RR (95% CI)	p-value	RR (95% CI)	p-value
ART	0.91 (0.79; 1.06)	0.25	0.96 (0.85; 1.09)	0.54
CD4 count (≤200 vs >200)	1.41 (1.23; 1.61)	<0.001	1.23 (1.08; 1.39)	0.001
Age	1.01 (0.99; 1.02)	0.23	0.99 (0.98; 1.01)	0.50
Gravidity	1.10 (1.01; 1.18)	0.02	1.07 (0.98; 1.16)	0.15
**Anaemia <72 hours after delivery**				
ART	1.05 (0.85; 1.29)	0.66	1.14 (0.93; 1.40)	0.22
CD4 count (≤200 vs >200)	1.38 (1.13; 1.68)	0.002	1.44 (1.17; 1.78)	0.001
Age	1.00 (0.99; 1.02)	0.64	1.00 (0.98; 1.03)	0.65
Gravidity	0.95 (0.86; 1.06)	0.38	0.93 (0.83; 1.05)	0.24
**Anaemia 2 weeks post delivery**				
ART	1.05 (0.85; 1.29)	0.66	1.14 (0.93; 1.40)	0.22
CD4 count (≤200 vs >200)	1.38 (1.13; 1.68)	0.002	1.44 (1.17; 1.78)	0.001
Age	1.00 (0.99; 1.02)	0.64	1.00 (0.98; 1.03)	0.65
Gravidity	0.95 (0.86; 1.06)	0.38	0.93 (0.83; 1.05)	0.24

## Discussion

Almost two thirds (64%) of the HIV positive pregnant women in our study were diagnosed with anaemia (HB<11 g/dL) in the 3rd trimester of pregnancy, and subsequently an additional 11.8% of the women were free from anaemia at screening but later developed anaemia during pregnancy or post-delivery. In total, 75.8% of the HIV positive women presented with anaemia (Hb<11 g/dL) during pregnancy and early postpartum. In a multivariable analysis, after adjusting for the antiretroviral regimen (ZDV or D4T/3TC/NVP), the lower CD4 count (<200 cells/mm^3^) remained a significant risk factor for anaemia during pregnancy and post-delivery. Moreover, having established that women in the advanced stage of HIV disease (CD4<200 cells/mm^3^) are more likely to be affected by anaemia in this study, we further report that there was no evidence of any added risk for anaemia with either of the ARV regimen (ZDV or the non ZDV containing triple ARV regimen) in women with a higher CD4 count.

Our study is one of few reported cohort studies of anaemia in HIV positive pregnant women. Similar to other reports, 60% of the anaemia cases in our study resolved post-delivery and the persistent anaemia cases post-delivery were more likely to have been moderate to severe microcytic hypochromic (iron deficiency) anaemia at baseline in pregnancy [15]. While we can attribute the resolution of anaemia to a combination of antiretroviral treatment and reversion to a non-pregnant physiological state, the persistent iron deficiency anaemia seems not to have been altered by standard haematinic supplementation during pregnancy. Identified as a limitation in our study, the data for this analysis are obtained at only 3 time points that includes 1 assessment only in the 3^rd^ trimester of pregnancy. Adherence to haematinic supplementation or duration of antiretroviral treatment was therefore not measured.

According to the WHO classification, the high occurrence of anaemia (HB<11 g/dL) in our cohort of HIV infected pregnant women (75.8%) would be considered a severe public health problem [16]. Although we had not sought to determine the independent role of HIV infection in the development of the high prevalence of anaemia, other studies in South Africa with an antenatal HIV prevalence of 30% and countries similarly affected by the HIV pandemic have confirmed that HIV infected adults are more likely to develop anaemia [17]. In the context of HIV infection, anaemia and iron deficiency have been often implicated as independent markers of HIV disease progression and mortality [18]–[19]. Further evidence of anaemia as a severe public health problem in South Africa is manifested in the recent South African maternal mortality report in which 9.2% of the maternal deaths were attributed to severe anaemia [8]. The report provides further evidence of severe anaemia being the more common cause of death among HIV infected women who were diagnosed with AIDS and did not receive ARV treatment (10%) as compared to women who were not eligible for ART (7%). Another study showed that women with severe anaemia at baseline had a 13 times greater risk of death during the first year of ART, further confirming that anaemia is strongly associated with HIV disease progression [20]. However whether anaemia is an outcome of or a cause of increased HIV virulence and immunological degradation requires further investigation.

Antiretroviral use, particularly ZDV has frequently and more recently been implicated in the development of anaemia in women and in their newborns [21]. Our study has not demonstrated such an association between either of the ARV regimens. In fact similar to a study in Tanzania, antiretroviral use (ZDV or D4T/3TC/NVP) in pregnancy may have prevented progression of anaemia as shown by the high resolution rate of anaemia (40%) and the higher median Hb level immediately postpartum and 14 day post-delivery [20]. Unlike the Tanzanian study though, most of the anaemia in our population were non-iron deficiency anaemia and more likely HIV associated with chronic infection and therefore resolving more rapidly with the use of ARV's in addition to the standard iron and folic acid micronutrient supplementation in pregnancy. In a study from Malawi, 16% of women with HIV had anaemia due to iron deficiency [22]. Only 14.5% of anaemia cases in our South African study population were related to iron deficiency, confirming that normocytic normochromic anaemia of chronic inflammation (due to reduced erythropoietin production) was the biggest contributor.

There were no maternal deaths in our cohort and neither was there a significant association between anaemia and adverse pregnancy outcomes. However, urinary tract infections were significantly more common among women with anaemia. Studies have implicated urinary tract infections in pregnancy as a cause of anaemia, women in our study who presented with low Hb levels at baseline were predisposed to urinary tract infections [23]. Other pregnancy complications and adverse pregnancy outcomes associated with anaemia such as pre-eclampsia, preterm labour, and low birth weight in other studies were not evidently associated with anaemia in our HIV infected pregnant population [24].

Although the association was not statistically significant (p = 0.09), infants born to mothers who presented with anaemia in pregnancy were twice more likely to be anemic (8.6% vs 3.8%) at birth. While evidence in some studies suggests that use of antiretrovirals in pregnancy were associated with haematological disorders in the newborns, our study also demonstrated a higher trend among infants born to mothers with CD4<200 and receiving ZDV but not among other mothers receiving triple ARVs [25]–[26]. Although in more recent studies, when HAART was rapidly becoming the standard of care for pregnant women, anaemia (grade 2 toxicity) was reported in almost half of the infant population and was most severe by 2 weeks of age [27]–[28]. Further studies are needed to explore the effect of ARVS on the neonatal outcomes.

A limitation of this study was that the sample was relatively small and was only powered to detect large differences between women with aneamia and without.

In conclusion, anaemia was most common among women in the advanced stage of HIV infection. There was no evidence of an association between ZDV or triple ARVs and anaemia. Antiretroviral treatment together with standard haematinic therapy could have contributed to the rapid resolution of anaemia post-delivery.

### Ethical Consideration

Institutional Regulatory (University of KwaZulu-Natal Biomedical Research Ethics Committee) was received for the HPTN046 study. Approval for this sub-study was also sought. All patient details for the sub-study will remain confidential, unique study participant numbers were used in data extraction. Written informed consent was obtained in the original study from eligible participants. No stored blood specimens were required in this sub study.
